# Red ginseng prevents doxorubicin-induced cardiomyopathy by inhibiting cell death via activating the Nrf2 pathway

**DOI:** 10.1186/s40959-024-00242-0

**Published:** 2024-06-22

**Authors:** Naoki Yoshikawa, Naoto Hirata, Yuichiro Kurone, Sadahiko Shimoeda

**Affiliations:** Department of Pharmaceutical Health Care and Sciences, Tokyo University of Pharmacy and Life Sciences, 1432-1 Horinouchi, Hachioji, Tokyo, 192-0392 Japan

**Keywords:** Doxorubicin, Anthracycline, Doxorubicin-induced cardiac dysfunction, Ginseng, Ginsenoside, Red ginseng, Nrf2, Ferroptosis, Apoptosis

## Abstract

**Background:**

Doxorubicin (DXR) is an effective chemotherapeutic agent. DOX-induced cardiomyopathy (DICM), a major limitation of DXR, is a complication with limited treatment options. We previously reported that Red Ginseng (steamed and dried the root of *Panax Ginseng* cultivated for over six years; RGin) is beneficial for the treatment of DICM. However, the mechanism underlying the action of RGin remains unclear. In this study, we investigated the mechanism of action underlying the efficacy of RGin in the treatment of DICM.

**Methods:**

Four-week-old DBA/2 mice were divided into: vehicle, DXR, RGin, and DXR + RGin (*n* = 10/group). Mice were treated with DXR (4 mg/kg, once a week, accumulated 20 mg/kg, i.p.) or RGin (0.5 g/kg, three times a week, i.p.). To evaluate efficacy, the survival rate and left ventricular ejection fraction (LVEF) were measured as a measure of cardiac function, and cardiomyocytes were subjected to Masson trichrome staining. To investigate the mechanism of action, western blotting was performed to evaluate the expression of nuclear factor erythroid 2-related factor 2 (Nrf2), heme oxygenase 1, transferrin receptor (TfR), and other related proteins. Data were analyzed using the Easy R software. Between-group comparisons were performed using one-way analysis of variance and analyzed using a post-hoc Tukey test. Survival rates were estimated using the Kaplan–Meier method and compared using the log-rank test. *P* < 0.05 was considered statistically significant in all analyses.

**Results:**

RGin treatment prolongs survival and protects against reduced LVEF. In the DXR group, Nrf2 was not activated and cell death was accelerated. Furthermore, there was an increase in the TfR levels, suggesting abnormal iron metabolism. However, the DXR + RGin group showed activation of the Nrf2 pathway and suppression of myocardial cell death. Furthermore, there was no increase in TfR expression, suggesting that there were no abnormalities in iron metabolism. Therefore, the mechanism of action of RGin in DICM involves an increase in antioxidant activity and inhibition of cell death through activation of the Nrf2 pathway.

**Conclusion:**

RGin is a useful therapeutic candidate for DICM. Its efficacy is supported by the activation of the Nrf2 pathway, which enhances antioxidant activity and inhibits cell death.

**Supplementary Information:**

The online version contains supplementary material available at 10.1186/s40959-024-00242-0.

## Background

Doxorubicin (DXR), an anthracycline antibiotic, was isolated in 1969 from the culture medium of a mutant *Streptomyces peucetius* strain [1]. DXR is a potent antineoplastic agent and exhibits strong anticancer activity against various types of cancer. Despite the recent emergence of various targeted molecular therapies, DXR remains a key drug in many therapeutic regimens [2]. However, the use of DXR is significantly limited by Doxorubicin-Induced Cardiomyopathy (DICM). DICM has long been recognized as a serious adverse complication and no fundamental treatment exists [3]. Unlike other cardiac diseases, DICM demands a unique approach owing to its poor prognosis, profound atrial and ventricular dilatation, and distinct pathological findings known as adria cells [3, 4]. Several cardioprotective agents have been proposed in clinical studies, but most have yielded negative results [3, 5]. This is partly because the mechanism of action of existing cardioprotective agents does not match that of DICM.

The mechanisms underlying DICM are complex and multifactorial, and a definitive understanding has not yet been established [3, 6, 7]. Among various hypotheses, reactive oxygen species (ROS) production is strongly supported as a major contributing factor to the pathogenesis of DICM [8, 9]. ROS production is exacerbated by factors such as oxygen, electrons, and abnormal iron metabolism [10, 11]. Specifically, cardiomyocytes that need to sustain an elevated exercise capacity exhibit a heightened requirement for oxygen and adenosine triphosphate. These essential molecules are predominantly generated through the mitochondrial electron transfer chain and tricarboxylic acid cycle. Notably, cardiomyocytes have an abundance of mitochondria that satisfy these metabolic demands. These processes significantly contribute to ROS production by generating abundant oxygen and electrons [12]. Additionally, cardiomyocytes require substantial amounts of iron to maintain muscle fibers, and iron can catalyze the production of potent ROS via the Fenton reaction with hydrogen peroxide [13]. Thus, cardiomyocytes are prone to ROS production, and, in particular, the generation of ROS due to abnormal iron metabolism may lead to ferroptosis, a form of cell death.

In particular, upon exposure to DXR, the quinone structure of DXR interacts with intracellular iron and oxygen, leading to its reduction and the formation of semiquinone DXR [14]. This process directly or indirectly promotes ROS production, possibly via Fenton’s reaction. To exacerbate this situation, a series of redox reactions are believed to occur repeatedly, generating substantial amounts of ROS from a single DXR molecule. Consequently, in cardiomyocytes exposed to DXR, the cyclic redox reactions of DXR play a crucial role in the increased generation of ROS and may induce ferroptosis and other forms of cell death [14, 15]. Therefore, agents with antioxidant or antiferroptotic properties are promising new therapeutic candidates.

Ferroptosis is a form of cell death caused by abnormal intracellular iron metabolism, which results in increased expression of the transferrin receptor (TfR). TfR plays a critical role in iron influx into cells, leading to elevated intracellular iron concentrations and accelerated ROS generation through the Fenton reaction [16]. In contrast, activation of the nuclear factor erythroid 2-related factor 2 (Nrf2) pathway is expected to suppress ferroptosis [17]. Nrf2 is a transcriptional regulator that responds to stress, and is typically maintained at a constant level in the cytoplasm via proteasomal degradation [18]. When activated by oxidative stress, Nrf2 evades ubiquitination and translocates to the cell nucleus, where it regulates the expression of various genes. These genes encompass those related to the antioxidant system and those involved in apoptosis suppression and inflammation [19–21]. Thus, many complex mechanisms underlying the pathogenesis of DICM may overlap.

We previously reported the efficacy of red ginseng (steamed and dried root of Panax Ginseng cultivated for over six years; RGin), which is safe, cost-effective [22]. And generally known to have antioxidant properties [23, 24]. However, we were not able to investigate this mechanism in our previous studies. Our previous report also showed interesting results: survival was prolonged when RGin was administered but declined after administration ceased, similar to the DXR-only group. This result indicates that the continued administration of RGin should be considered. Therefore, this study aimed to explore the mechanism of action of RGin using our chronic DICM model, focusing on the Nrf2 pathway and cell death. We further decided to study survival when RGin was continued after the end of DXR treatment.

## Methods

### Animals

Four-week-old male DBA/2 mice were obtained from Japan SLC, Inc. All mice were acclimated in a pathogen-free animal facility for six days after purchase. Each mouse was randomly assigned to one of four groups: vehicle (*n* = 10), DXR (*n* = 10), RGin (*n* = 10), or DXR + RGin (*n* = 10). Furthermore, for the purpose of examining survival, an independent experimental system was constructed under exactly the same conditions: DBA/2 mice were purchased from the same supplier, and each mouse was randomly assigned to one of four groups: vehicle (*n* = 10), DXR (*n* = 11), RGin (*n* = 10), or DXR + RGin (*n* = 10). The average weight of the animals at the beginning of the experiment was 18.0 g. All mice were maintained on a 12-h light/dark cycle and had ad libitum access to food and water. The number of animals was determined in accordance with the ARRIVE guidelines, and a preliminary statistical analysis was performed to establish the minimum number of animals required [25]. A significance level (α) of 0.05, statistical power of 0.8, expected mean difference of 10.5 between groups, and standard deviation of 8.35. Two-tailed tests were conducted. The sample size for the analysis was 10 animals. These statistical parameters were based on those used in previous experiments. We designed our experiment based on these statistical results.

### Ethics

All the experiments and animal care were conducted in accordance with the principles of Good Laboratory Practice. The experimental animals were handled and treated in strict compliance with the regulations for handling experimental animals at the Tokyo University of Pharmacy and Life Sciences. This study was approved by the Experimental Animal Committee (Permit No: P22-50).

### Treatment

Drugs were administered as previously described [22]. DXR (4 mg/kg) was administered intraperitoneally on days 2, 9, 16, 25, and 32, at a cumulative dose of 20 mg/kg. Similarly, RGin (500 mg/kg) was intraperitoneally administered three times a week for a total of 15 days (days 1, 3, 5, 8, 10, 12, and 35). The vehicle was administered with the same volume of saline solution as the DXR or RGin at the corresponding time points.

Additionally, the DXR + RGin and RGin groups assigned to the survival analysis received DXR or saline for 5 weeks, followed by RGin three times a week until day 84 at the end of the observation period.

### Drugs

DXR (10 mg/5 mL) was purchased from Sandoz Co. (Basel, Switzerland). The RGin injection was formulated using ginseng powder purchased from Tsumura Co. (Tokyo, Japan). The saline solution was purchased from Otsuka Pharmaceutical Co. (Tokyo, Japan). Ethanol (99.5% purity), butorphanol, medetomidine, and midazolam were purchased from Fujifilm Wako Pure Chemical Co. (Osaka, Japan).

### Preparation of RGin injection solution

The RGin extraction method, as described in our previous study [22], involved dissolving 5 g of RGin powder in 100 mL of 50% ethanol, followed by boiling to purify the extract. After centrifugation at 1000 g for 45 min, the supernatant was separated and lyophilized. The dried material was then reconstituted with 10 mL of saline and filtered to create a 500 mg/mL solution for RGin injection. Previous component analyses using liquid chromatography with tandem mass spectrometry indicated no significant differences compared to common extraction methods [22, 26]. This analysis confirmed that the current dosage was neither excessive nor underestimated.

### Sample collection

The sample-collection protocol complied with the guidelines and regulations of the Animal Experiment Committee. First, echocardiography examinations were performed under general anesthesia using a combination of butorphanol (5 mg/kg), medetomidine (0.75 mg/kg), and midazolam (4 mg/kg). Mice were euthanized using carbon dioxide gas after echocardiography. Second, the abdomen was opened using dissecting scissors and the heart was removed for tissue preparation and protein analysis.

### Survival analysis

Survival of DBA/2 mice (*n* = 41) was monitored for 12 weeks. The occurrence of an event was defined as death and was evaluated by recording the number of mice that died and the number of days of survival.

### Echocardiography

Echocardiographic examinations were performed at 1, 3, 5, and 7-week time points. Butorphanol, medetomidine, and midazolam were intraperitoneally administered to mice under general anesthesia. Unnecessary chest hair was removed using an electric shaver after confirming the disappearance of the orthorectal reflex. An M-mode echocardiogram was obtained using a Sonoscape SV6 7–4 MHz probe (SonoScape Medical Corp., Shenzhen, China). The left ventricle in the image was assumed to be a rotating ellipsoid. The vertical distance from the endocardial surface of the left ventricular wall to the posterior wall of the left ventricle was measured on the short-axis image in the beam direction, passing through the largest short diameter of the left ventricle at end-diastole and end-systole. The end-diastolic (left ventricular end-diastolic diameter; LVDd) and end-systolic (LVDs) diameters were determined based on these measurements. left ventricular ejection fraction (LVEF) was calculated using the Teichholz method. This method involves calculating the left ventricular end-diastolic volume (LVEDV) and left ventricular end-systolic volume (LVESV), followed by fractional shortening (FS) and LVEF as follows:

$$\text{FS}\ ({\%}) = \frac{\left(\text{LVDd}-\text{LVDs}\right)}{\text{LVDd}}\times 100$$

$$\text{LVEF}\ (\%) = \frac{\text{LVEDV} - \text{LVESV}}{\text{LVEDV}}\times 100$$

### Iron assay

An Iron Assay Kit (Metallogenics, Japan) was used to measure the total iron concentration in the cardiomyocytes at 1, 3, 5, and 7-week time points. This kit is based on the ferrozine chromogenic method. The total iron concentration in cardiomyocytes was measured according to the manufacturer’s protocol. Samples or standards were mixed with buffer and absorbance (OD1) was measured at 560 nm. The chromogenic agent was then added to all the wells, and the absorbance (OD2) was measured again at 560 nm. The final iron concentration was calculated using the following equation: All assays were performed using at least three independent measurements.

$$\text{Iron concentration}\left(\upmu{g}/\text{dL}\right)=\frac{\text{OD2}_{\text{sample}}-\text{OD1}_{\text{sample}}}{\left(\text{OD2}_{\text{standard}}-\text{OD1}_{\text{standard}}\right)}\times{200}$$

### Histological evaluation of cardiomyocytes

The hearts were washed with saline and immediately fixed in 10% neutral-buffered formalin solution (pH 7.4) after appropriate removal and separation using dissecting scissors. Fixed tissues were transported to an external laboratory (Biopathology Institute Co., Ltd., Oita, Japan) and embedded in paraffin. Sections were prepared from tissues sliced from the aortic valve. Finally, the tissues were subjected to Masson's trichrome (MT) staining and transferase dUTP nick-end labeling (TUNEL) staining. Images obtained by TUNEL staining were subjected to quantitative analysis using ImageJ (Ver. 1.53). Magnification was fixed for all images. Three different image fields of view were extracted from each specimen and these sites were clipped from similar locations across all specimens. Histopathological evaluation was performed using Olympus Net Image Server SQL (Olympus Co., Ltd., Tokyo, Japan).

### Western blotting

Explanted hearts at 7-weeks were chemically homogenized using a Nuclear Extract kit (Active Motif, Inc. US). After homogenization, the cytoplasmic and nuclear fractions were extracted according to the prescribed protocol. Each extracted fraction was subjected to protein quantification using the Bradford method with bovine serum albumin as the standard. Sample proteins (20 μg) were separated by sodium dodecyl sulfate–polyacrylamide gel electrophoresis (SDS-PAGE) using u-PAGEL H 4–20% (ATTO Co, Japan). After SDS-PAGE, Powered BLOT 2 M (ATTO Co., Japan) was used at 12 V for 45 min to transfer the proteins from the gel to a nitrocellulose membrane.

The membrane was soaked in 1 × iBind Flex Solution formulated using the iBind Flex Solution Kit (SLF2020, Invitrogen). An iBind Flex Card (SLF2010, Invitrogen) was placed on the iBind Flex device and soaked in 1 × iBind Flex Solution. Later, the soaked membrane was placed onto the soaked iBind Flex Card and rolled with a roller to ensure full contact with the iBind Flex Card. Subsequently, primary and secondary antibody solutions were formulated with 1 × iBind Flex Solution and added to the corresponding antibody tank according to the instructions provided with the iBind Flex Western device. See Table 1 for the origin and dilution of each antibody. After completion of device operation, the device was kept running for 2.5 h or more to allow for sufficient antibody incubation and washing. Following the completion of the run of the device, the membrane was fully rinsed with purified water. Chemiluminescence image was obtained by LAS-3000 (FujiFilm Co, Japan), with using ECL Select™ Western Blotting Detection Reagent (Cytiva, Japan). The obtained images were analyzed using the ImageJ software (Ver 1.53). All analyses obtained using ImageJ software were illustrated semi-quantitatively with reference to housekeeping proteins in the vehicle group.

**Table 1:** List of antibodies used for Western blotting

Target	Primary or Secondary	Source	Molecular Weight	Dilution	Company
Nrf2	Primary	rabbit	97–100	1: 1,000	Cell Signaling Technology, Inc., USA
HO-1	Primary	rabbit	28	1: 1,000	Cell Signaling Technology, Inc., USA
Transferrin Receptor (CD71)	Primary	rabbit	85	1: 1,000	Bioss, Inc., USA
β-actin	Primary	rabbit	45	1: 1,000	Cell Signaling Technology, Inc., USA
Histone H2B	Primary	mouse	18	1: 1,000	Santa Cruz Biotechnology, Inc., USA
Peroxidase-conjugated AffiniPure Goat Anti-mouse IgG	Secondary	goat	―	1: 20,000	Jackson ImmunoResearch Labolatories, Inc., USA
Rabbit IgG Horseradish peroxidase-conjugated Antibody	Secondary	goat	―	1: 1,000	Cell Signaling Technology, Inc., USA

### Statistical analyses

All data were analyzed using Easy R (EZR) version 1.55 [27]. Between-group comparisons were performed using one-way analysis of variance and analyzed using a post-hoc Tukey test. Survival rates were estimated using the Kaplan–Meier method and compared using the log-rank test. *P* < 0.05 was considered statistically significant in all analyses.

## Results

### Efficacy of RGin for DICM

**Fig. 1:**
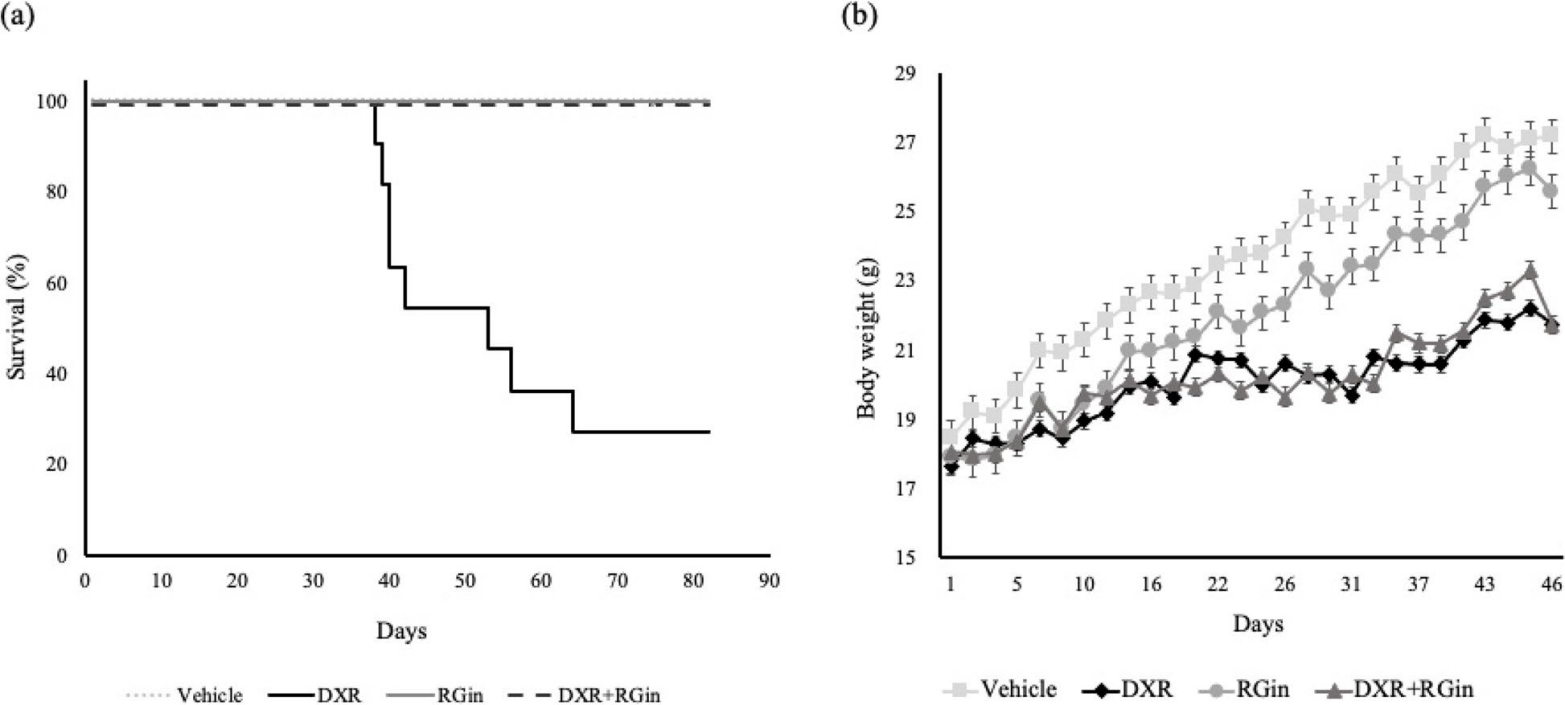
Physiological Evaluation for DICM. **a** Kaplan–Meier curves, log-rank test *p* < 0.05. **b** Trend of body weight

Following DXR administration, a marked increase in cell death was observed in the DXR group. In contrast, survival was significantly prolonged in the DXR + RGin (*P* < 0.05; Fig. 1a). However, weight loss was observed in both DXR and DXR + RGin groups (Fig. 1b).

**Fig. 2:**
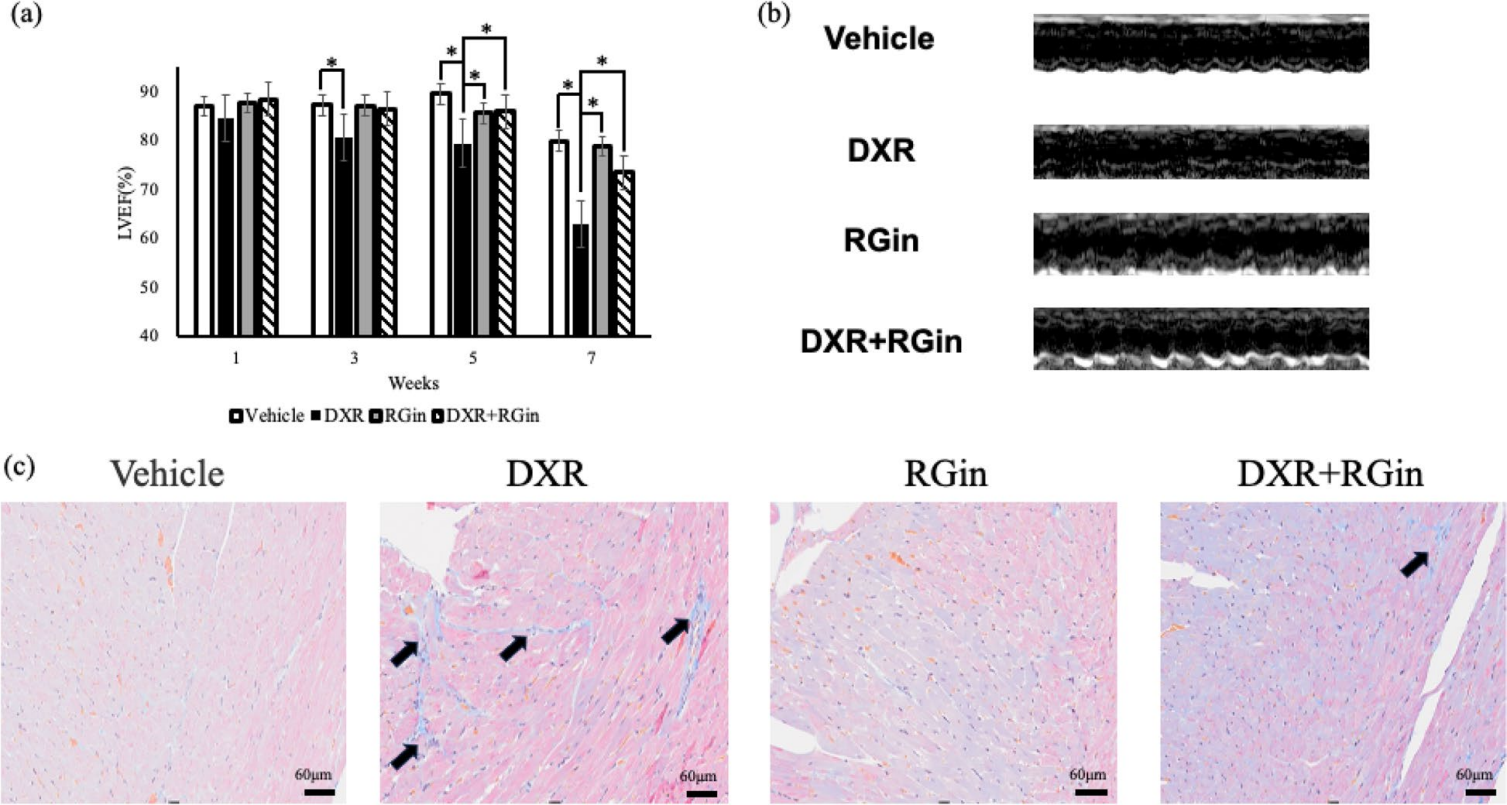
Functional and histological evaluation of the heart. **a** LVEF **b** M-mode echocardiographic images **c** MT staining. LVEF: left ventricular ejection fraction, MT: masson trichrome. *P < 0.05 was considered a significant difference

Echocardiography revealed a significant decrease in the LVEF in the DXR-treated group. The decrease in LVEF began approximately three weeks after the start of DXR administration and persisted even after drug withdrawal. The DXR + RGin group consistently maintained a significantly higher LVEF than the DXR group. Similarly, there was no reduction in LVEF in the RGin alone group (Fig. 2a). Other echocardiography results showed significant increases in the LVDs and LVESV in the DXR group at week 7 (Table 2). MT staining revealed fibrosis in the DXR group, which was reduced by the co-administration of RGin (Fig. 2c).

**Table 2:** Results of echocardiography

	Vehicle ± SD	DXR ± SD	RGin ± SD	DXR + RGin ± SD
**1 Week**				
LVDd (mm)	2.55 ± 0.542	2.51 ± 0.240	2.79 ± 0.903	2.21 ± 0.501
LVDs (mm)	1.15 ± 0.323	1.19 ± 0.181	1.23 ± 0.442	0.960 ± 0.223
LVEDV (μL)	25.3 ± 14.4	22.9 ± 5.72	34.6 ± 30.1	17.9 ± 10.8
LVESV (μL)	3.54 ± 2.68	3.47 ± 1.43	4.59 ± 4.57	2.07 ± 1.31
FS (%)	55.3 ± 5.74	52.3 ± 7.21	56.3 ± 4.96	56.5 ± 4.83
LVEF (%)	87.1 ± 4.77	84.6 ± 5.96	87.8 ± 4.27	88.4 ± 3.72
**3 Week**				
LVDd (mm)	2.99 ± 0.346	2.80 ± 0.797	2.95 ± 0.322	2.53 ± 0.690
LVDs (mm)	1.32 ± 0.323	1.46 ± 0.449	1.30 ± 0.262	1.15 ± 0.331
LVEDV (μL)	35.4 ± 9.57	33.2 ± 21.5	34.3 ± 8.93	25.9 ± 16.0
LVESV (μL)	4.79 ± 2.62	6.62 ± 5.05	4.49 ± 2.38	3.50 ± 2.69
FS (%)	56.5 ± 7.20*	47.9 ± 5.04	56.1 ± 6.69*	54.4 ± 5.21
LVEF (%)	87.4 ± 5.02*	80.7 ± 4.98	87.2 ± 4.85*	86.6 ± 3.95*
**5 Week**				
LVDd (mm)	3.20 ± 0.410	3.03 ± 0.793	3.33 ± 0.511	3.23 ± 0.542
LVDs (mm)	1.26 ± 0.343	1.02 ± 0.501	1.52 ± 0.306	1.46 ± 0.371
LVEDV (μL)	42.1 ± 14.0	39.8 ± 24.6	46.9 ± 17.1	43.8 ± 17.2
LVESV (μL)	4.42 ± 2.68	8.51 ± 7.06	6.99 ± 4.56	6.40 ± 4.68
FS (%)	60.8 ± 9.79*	47.5 ± 7.95	54.5 ± 7.38	54.9 ± 6.91*
LVEF (%)	89.6 ± 6.42*	79.5 ± 8.56	85.6 ± 5.24*	85.9 ± 5.81*
**7 Week**				
LVDd (mm)	2.86 ± 0.621	3.25 ± 0.662	2.87 ± 0.521	3.12 ± 0.762
LVDs (mm)	1.52 ± 0.431*	2.17 ± 0.562	1.55 ± 0.511*	1.82 ± 0.551*
LVEDV (μL)	33.5 ± 18.2	45.5 ± 20.6	33.5 ± 15.8	42.1 ± 27.2
LVESV (μL)	7.04 ± 6.66*	17.8 ± 10.6	8.03 ± 8.18*	11.8 ± 9.90*
FS (%)	47.8 ± 7.97	33.7 ± 7.93	47.2 ± 9.63	41.9 ± 7.73
LVEF (%)	80.0 ± 8.02*	62.9 ± 11.3	78.9 ± 10.2*	73.5 ± 9.15*

All data were analyzed using one-way analysis of variance and Tukey’s post-hoc test. Asterisks indicate statistically significant results compared to the DXR group; *P < 0.05

### Action of RGin on the Nrf2 pathway

**Fig. 3:**
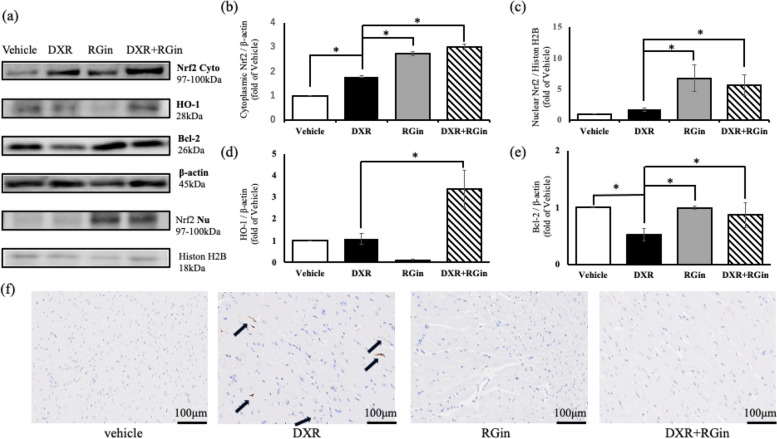
Evaluation of Nrf2 pathway by western blotting in cardiomyocytes at week 7. **a** Bands of western blotting. **b** Nrf2 expression levels in cytoplasm. **c** Nrf2 expression levels in the nucleus. **d** HO-1 expression levels **e** Bcl-2 expression levels. **f** TUNEL staining in cardiomyocytes. *P < 0.05 was considered significantly different

To elucidate the mechanism of action of RGin, we examined the expression levels of proteins associated with the Nrf2 pathway in cardiomyocytes at seven 7-week. Cytoplasmic Nrf2 expression was significantly higher in all groups than in the vehicle group (Fig. 3b). However, nuclear Nrf2 expression was detected only in the RGin and DXR + RGin groups (Fig. 3c). Furthermore, we observed increased expression of heme oxygenase 1 (HO-1), an Nrf2 target gene known for its antioxidant properties, only in the DXR + RGin group (Fig. 3d). The expression level of B-cell lymphoma 2 (Bcl-2), a target gene of Nrf2 and an anti-apoptotic agent, significantly decreased only in the DXR group (Fig. 3e). In contrast, Bcl-2 expression was improved with RGin administration. Indeed, TUNEL staining detected a large number of TUNEL-positive cells in the DXR group and almost no TUNEL-positive cells in the DXR + RGin group (Fig. 3f). The analysis yielded the following TUNEL positivity rates per field of view: vehicle, 0.00334%; DXR, 0.335%; RGin, 0.0139%; and DXR + RGin, 0.0351%. The DXR group showed a significantly higher TUNEL positivity rate than the other three groups.

### Effects of RGin on *iron* in vivo

**Fig. 4:**
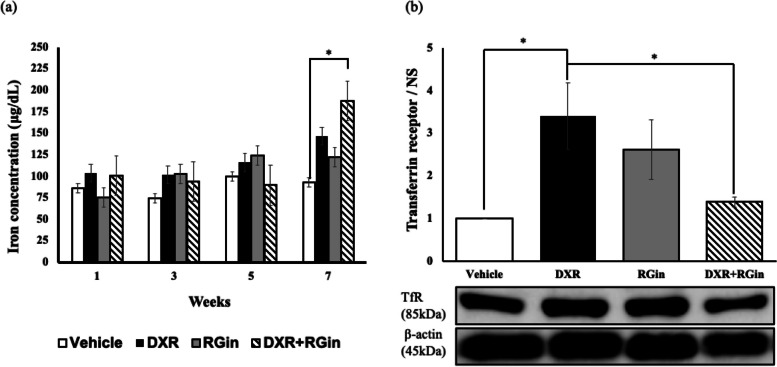
Effects of RGin on iron in vivo. **a** Iron concentration in cardiomyocytes by immunological assay. **b** Evaluation of TfR expression by western blotting in cardiomyocytes at week 7. *P < 0.05 was considered significantly different

Throughout the drug treatment period (up to week 5), myocardial intracellular iron concentration did not exhibit significant changes. At week 7, intracellular iron concentration showed a notable increase in the DXR group, although this difference was not statistically significant. Conversely, a significant increase was observed in the DXR + RGin group compared to the vehicle group (Fig. 4a). TfR expression in the cardiomyocytes at week 7 was significantly higher in the DXR group. In contrast, the DXR + RGin group exhibited a slight increase in TfR expression (Fig. 4b).

## Discussion

This study aimed to investigate the efficacy of RGin in chronic DICM mouse models and to clarify its mechanism of action. Several compounds, including ginsenosides, have been reported to be effective against DICM. However, most of these studies were based on acute DICM models, and need to be considered to determine whether they reflect the clinical features of chronic DICM. The model adopted in this study was based on the DXR administration method proposed by Sabatino et al., and the strains were determined in our previous studies [7, 22, 28].

In this study, long-term survival observations revealed a decreased survival rate over time in the DXR group and a significantly improved survival rate in the DXR + RGin group. These results indicate that RGin can reduce the lethal toxicity of DXR and demonstrate the utility of continued RGin treatment, even after the completion of DXR treatment. We hypothesized that DBA/2 mice may be less tolerant to cardiotoxic compounds compared to the other strains. This reduced tolerance is likely due to genetic factors such as congenital epicardial calcification and mutations in Myh7 and Mybpc3. Owing to these genetic characteristics, DBA/2 mice are a suitable mouse model for hypertrophic cardiomyopathy [29]. Furthermore, previous studies examining CAWS-induced myocarditis in several strains have shown low survival rates only in DBA/2 [28]. These findings support our results of early and low survival rates following DXR administration. Echocardiography showed a reduced LVEF in the DXR group 3 weeks after DXR administration. In our model, the DXR-induced decrease in LVEF was irreversible despite discontinuation of DXR. In addition, some echocardiography results showed that decreased contractility did not decrease the diastolic capacity. In contrast, the DXR + RGin group showed no significant reduction in LVEF during any of the observation periods, although weight loss was not suppressed. These findings of this model reflect the clinical features of DICM and strongly support RGin as a potential therapeutic candidate for DICM. We hypothesize that the cardiac fibrosis observed with MT staining is due to RGin regulated Several molecules such as Nrf2 and transforming growth factor β (TGF-β). In general, fibrosis is explained by ROS activation, enhanced inflammatory responses, and collagen overaccumulation. Our results showed enhanced activation of Nrf2 and its antioxidant activity. Furthermore, several studies indicate that RGin inhibits collagen hyperaccumulation by suppressing TGF-β expression [30, 31].

The essential question of This study aimed to investigate the mechanism of action of RGin in DICM. The involvement of ROS in DICM pathogenesis has been strongly suggested [8, 9]. Under these pathological conditions, certain ginsenosides have antioxidant properties, making them promising therapeutic agents for DICM [32, 33]. Ginsenoside Rg1 is effective in DICM by reduce the expression of P62 and ATG5 (E3 ubiquitin-like ligase) and inhibiting autophagy [34]. Furthermore, a mixed Chinese herbal injection consisting of *Panax ginseng* and Ophiopogon japonicus has been reported to inhibit the release of inflammatory cytokines such as interleukin -6, avoiding the inflammation of cardiomyocytes and DICM [35, 36]. However, there is no consensus on the molecular mechanisms of RGin in DICM. This is because previous reports were based on the acute DICM model, which may not accurately reflect the pathophysiology. For this reason, the mechanisms underlying its long-term efficacy are poorly understood. In this study, we focused on the Nrf2 pathway in a mouse model of chronic DICM. This pathway is associated with antioxidant activity and cell death, and is strongly implicated in the pathogenesis of DICM.

The results of western blotting showed that DXR + RGin induced Nrf2 activation and promoted the expression of HO-1 and Bcl2. These molecules contribute to the antioxidant effects and inhibition of apoptosis [37, 38]. The antioxidant effect of RGin can be attributed to an increase in HO-1 expression through the activation of Nrf2 [39–41]. Nrf2 is located upstream of cell death-related signaling and suppresses multiple cell death pathways by regulating the expression of its target molecules [42].

In particular, ferroptosis is thought to be a major cause of cell death in DICM based on the histopathological characteristics of cardiomyocytes and the structural features of DXR [14, 43]. Fang et al. treated DICM mice with several specific cell death inhibitors, including ferroptosis, apoptosis, and autophagy, and found that ferroptosis contributed the most [44]. However, it is difficult to determine whether ferroptosis occurs as specific molecular markers have not yet been identified [45, 46].

Therefore, we also investigated iron levels in cardiomyocytes and TfR. Iron and TfR levels are often used in ferroptosis studies [16]. There was no consistent association between iron levels in cardiomyocytes and TfR expression in this study; the DXR group showed an increase in iron levels and TfR expression, although the increase in iron concentration was not significant. In contrast, the DXR + RGin group showed a significant increase in iron levels but suppressed TfR expression. In general, TfR is considered a representative indicator with high specificity for ferroptosis. In contrast, it has been proposed that intracellular iron levels may not necessarily contribute to the promotion of ferroptosis. Various research groups, especially the pioneers in the concept of ferroptosis, have argued that elevated intracellular iron concentrations do not necessarily trigger ferroptosis [47, 48]. Our results did not contradict this hypothesis. This suggests that an increase in intramyocardial iron concentration may not be consistent with the progression of ferroptosis and DICM.

**Fig. 5:**
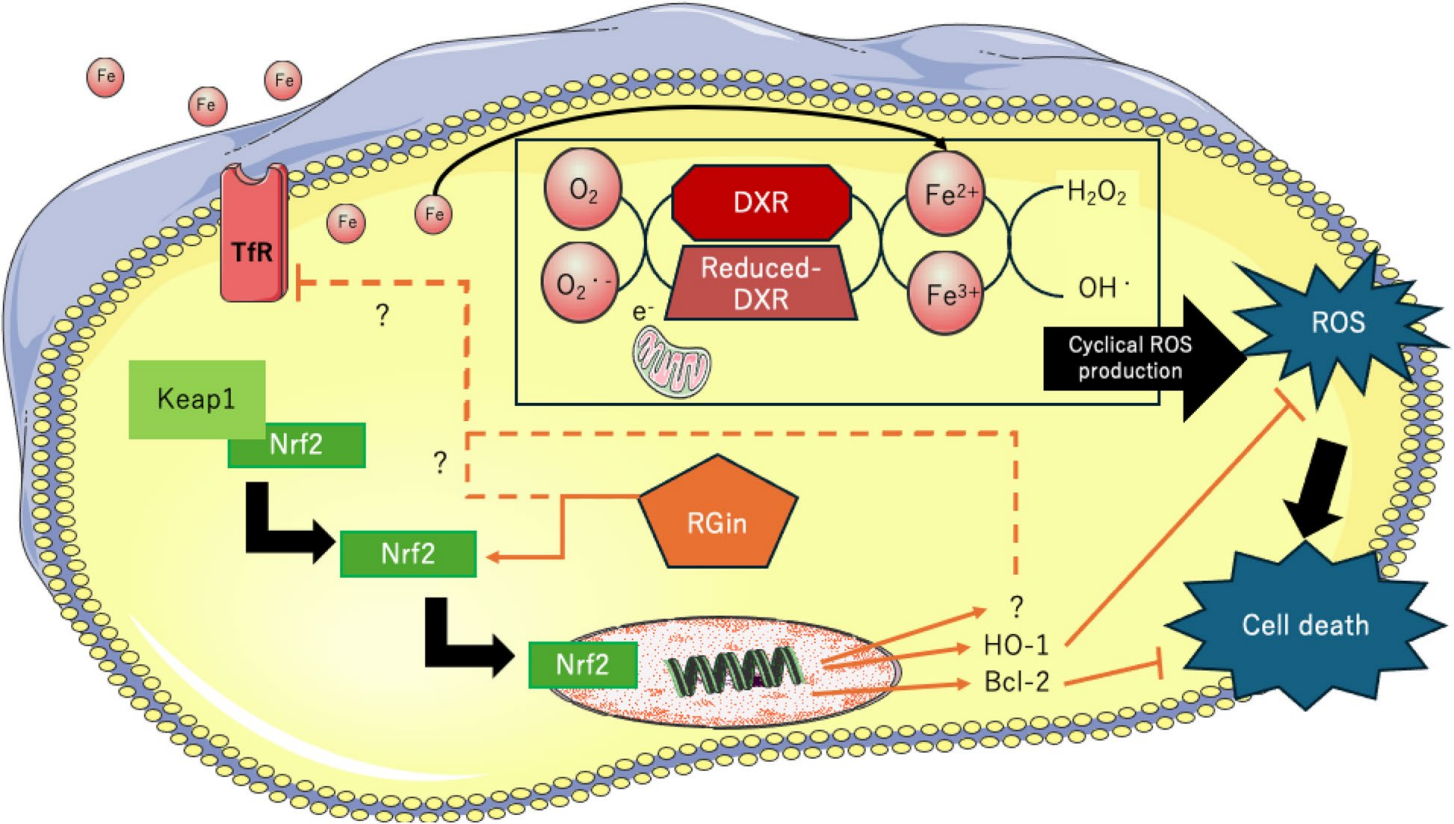
Proposed mechanism of RGin for DICM in this study. This figure was drawn using pictures from Server Medical Art. Servier Medical Art by Servier is licensed under a Creative Commons Attribution 4.0 Unported License

Our results that increase of total intracellular iron in the DXR + RGin group with preserved LVEF can consider from intracellular iron "status.” Free Fe^2+^ contributes to ferroptosis that is not bound to iron-binding proteins such as ferritin and heme [49–51]. However, this study only examined the total amount of iron and did not distinguish between Fe^2+^, Fe^3+^, and protein binding. In contrast, some studies have reported that Nrf2 activation promoted ferritin and heme production and our results showed that RGin treatment activated the Nrf2 pathway [52, 53]. Based on our results and these reports, we propose a mechanism of RGin in DICM, suggesting the following hypothesis regarding Nrf2 and ferroptosis (Fig. 5). The administered DXR interacted with iron to accelerate cyclic ROS production, which induced TfR expression and increased intracellular iron concentration. In contrast, co-administration of RGin promoted ferritin and heme synthesis through the activation of Nrf2. This increases the proportion of protein-bound iron to free Fe^2+^. Protein-bound iron did not contribute to increased TfR expression. This suggests that co-administration of DXR and RGin increases the proportion of protein-binding iron and prevents ferroptosis. However, in the context of DICM, Nrf2-ferroptosis has not been sufficiently studied in the context of DICM [43]. This study provided several insights into the relationship between DICM and Nrf2.

Our study had some limitations. First, this study was not performed using a mouse model of cancer. Further, we did not examine whether RGin reduced the anti-tumor activity of DXR. Although RGin is generally recognized as an agent that enhances resistance to tumor activity, no studies have simultaneously examined the inhibition of DICM development and avoidance of reduced antitumor activity [54, 55]. This is the next research question. Second, we did not investigate all signaling molecules associated with Nrf2, ferroptosis, or apoptosis. Our study selectively focused on the representative signals of each factor, although our results revealing Nrf2 activation contributing to the suppression of ferroptosis and apoptosis. Nevertheless, these findings are not sufficiently comprehensive to fully elucidate the intricate molecular networks in vivo. Additionally, the pathogenesis of DICM remains poorly understood. To address this issue, we will continue our investigation using qPCR and next-generation sequencers.

In summary, this study demonstrates the effectiveness of RGin treatment in alleviating chronic DICM, further strengthening the potential of RGin as a therapeutic option for DICM. This study underscores the vital role of curtailing cell death processes, such as ferroptosis and apoptosis, through the transcriptional regulation of target molecules associated with Nrf2 activation as the fundamental mechanism behind the efficacy of RGin. Finally, while the direct association of RGin with the inhibition of ferroptosis is not explicit, it sheds light on the target molecules of Nrf2 and iron kinetics. These findings significantly contribute to an improved understanding of DICM pathogenesis and offer potential targets for attenuating DICM.

## Conclusion

RGin has demonstrated significant efficacy against chronic DICM. Its mechanism is associated with the inhibition of cell death through the activation of the Nrf2 pathway.

### Supplementary Information

Supplementary Material 1.

## Data Availability

No datasets were generated or analysed during the current study.
