# Supplementary figures and images for: Red ginseng prevents doxorubicin-induced cardiomyopathy by inhibiting cell death via activating the Nrf2 pathway
Source: Cardiooncology. 2024 Jun 22;10:39. doi: 10.1186/s40959-024-00242-0 (PMC11193215; doi:10.1186/s40959-024-00242-0)

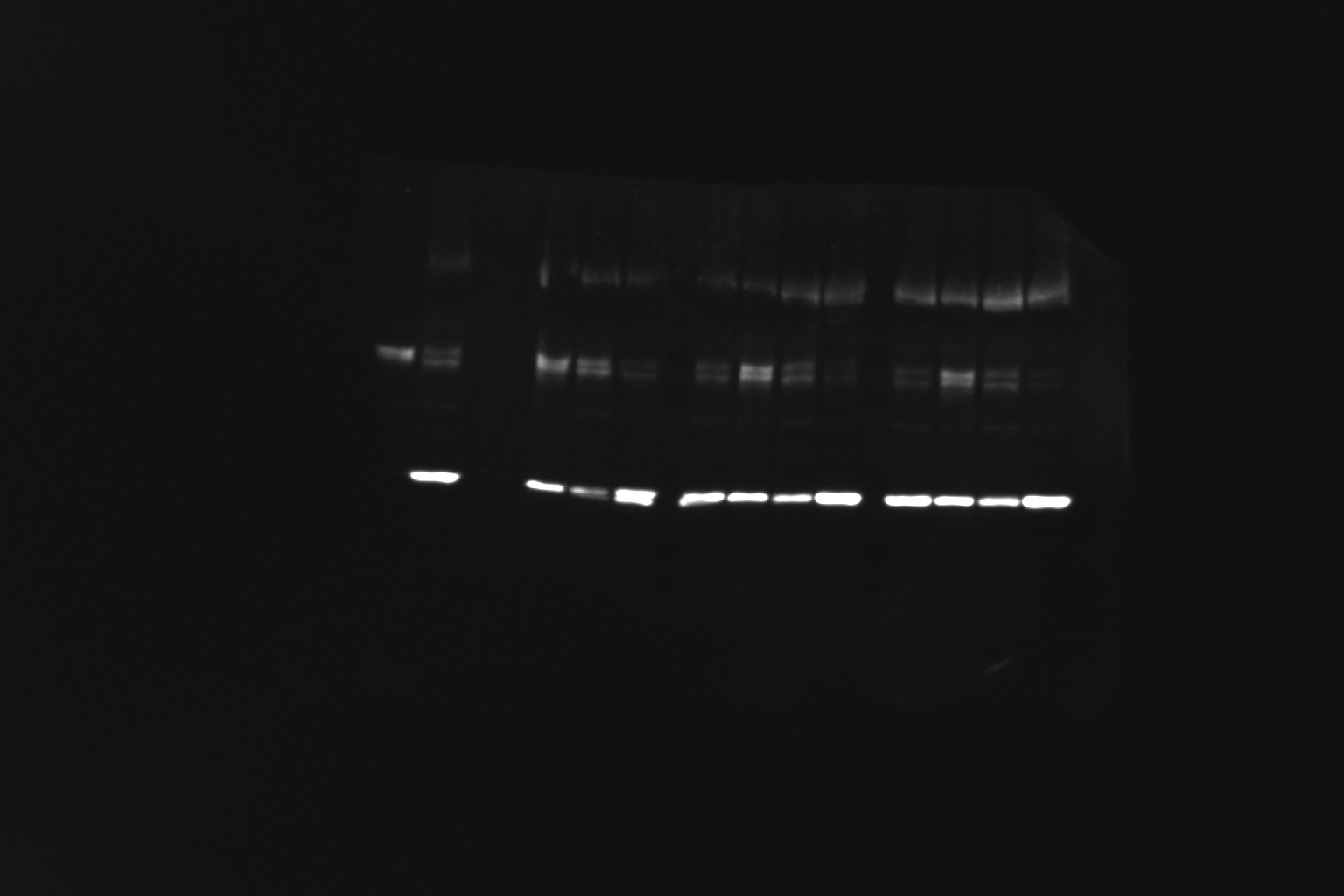

Supplement: Supplementary file 1 — Supplementary Material 1. [file 40959_2024_242_MOESM1_ESM.zip › actin(Nrf2).TIFF]

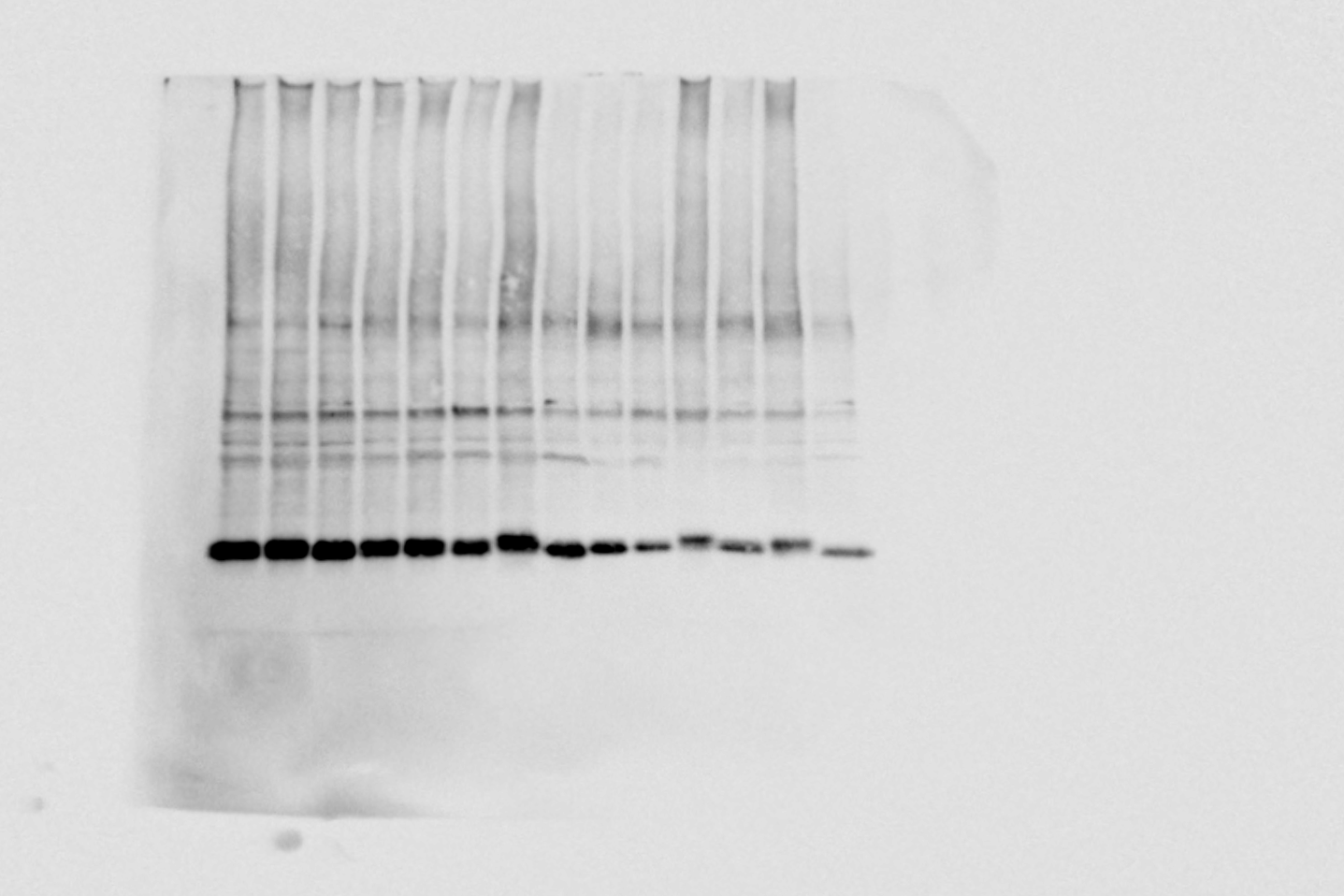

Supplement: Supplementary file 1 — Supplementary Material 1. [file 40959_2024_242_MOESM1_ESM.zip › actin.revision.tiff]

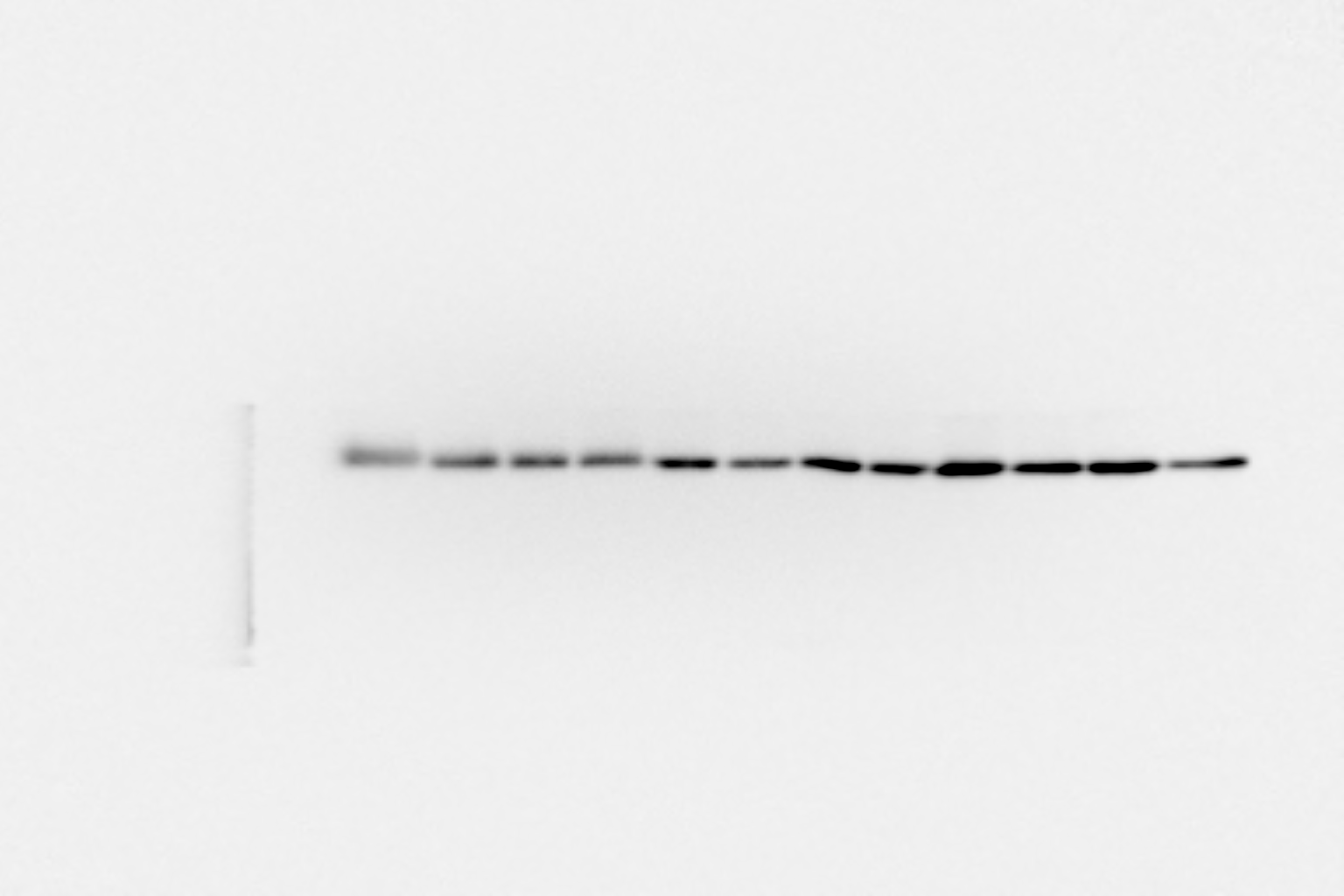

Supplement: Supplementary file 1 — Supplementary Material 1. [file 40959_2024_242_MOESM1_ESM.zip › Bcl2.TIFF]

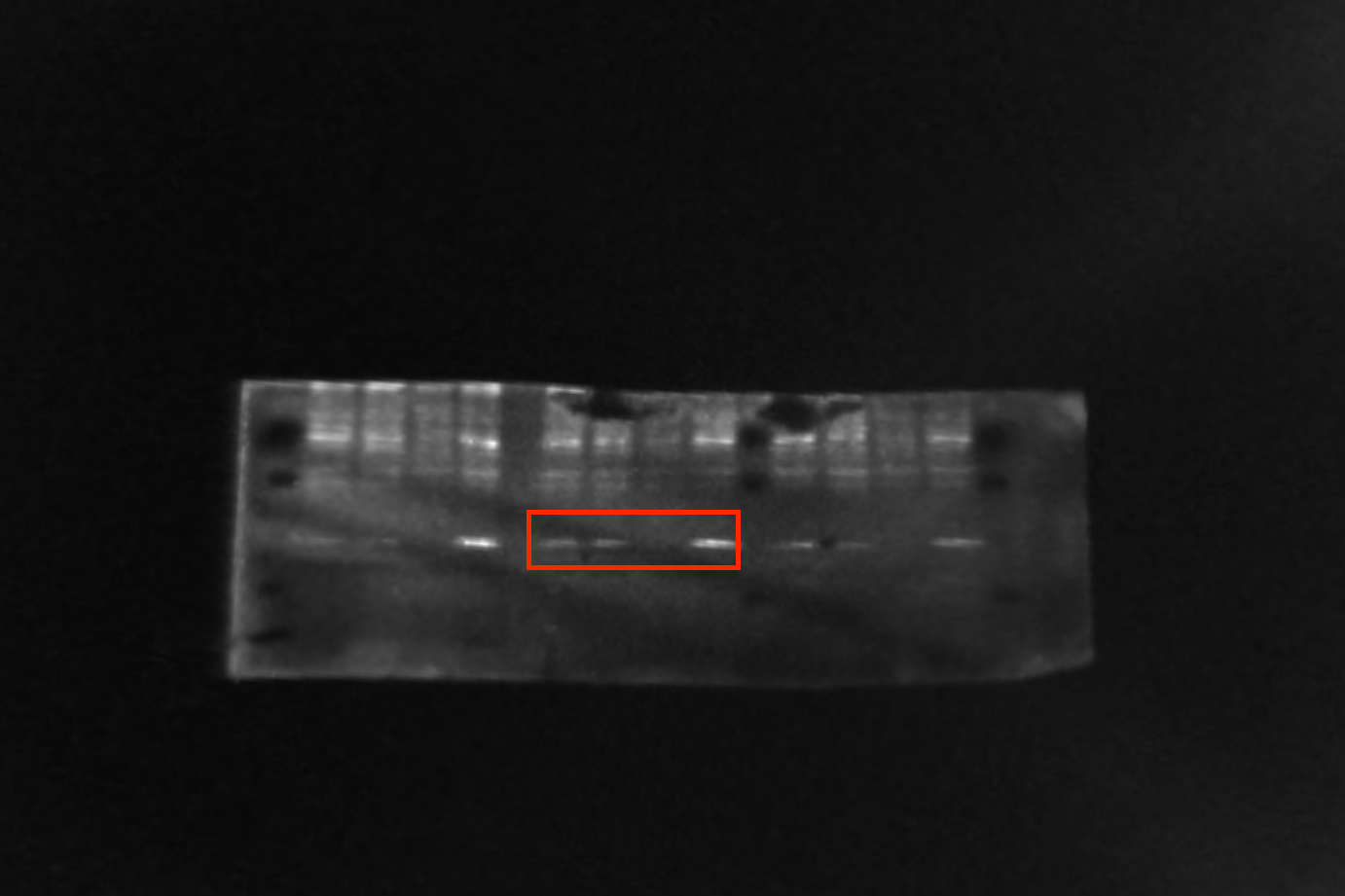

Supplement: Supplementary file 1 — Supplementary Material 1. [file 40959_2024_242_MOESM1_ESM.zip › HO1.tiff]

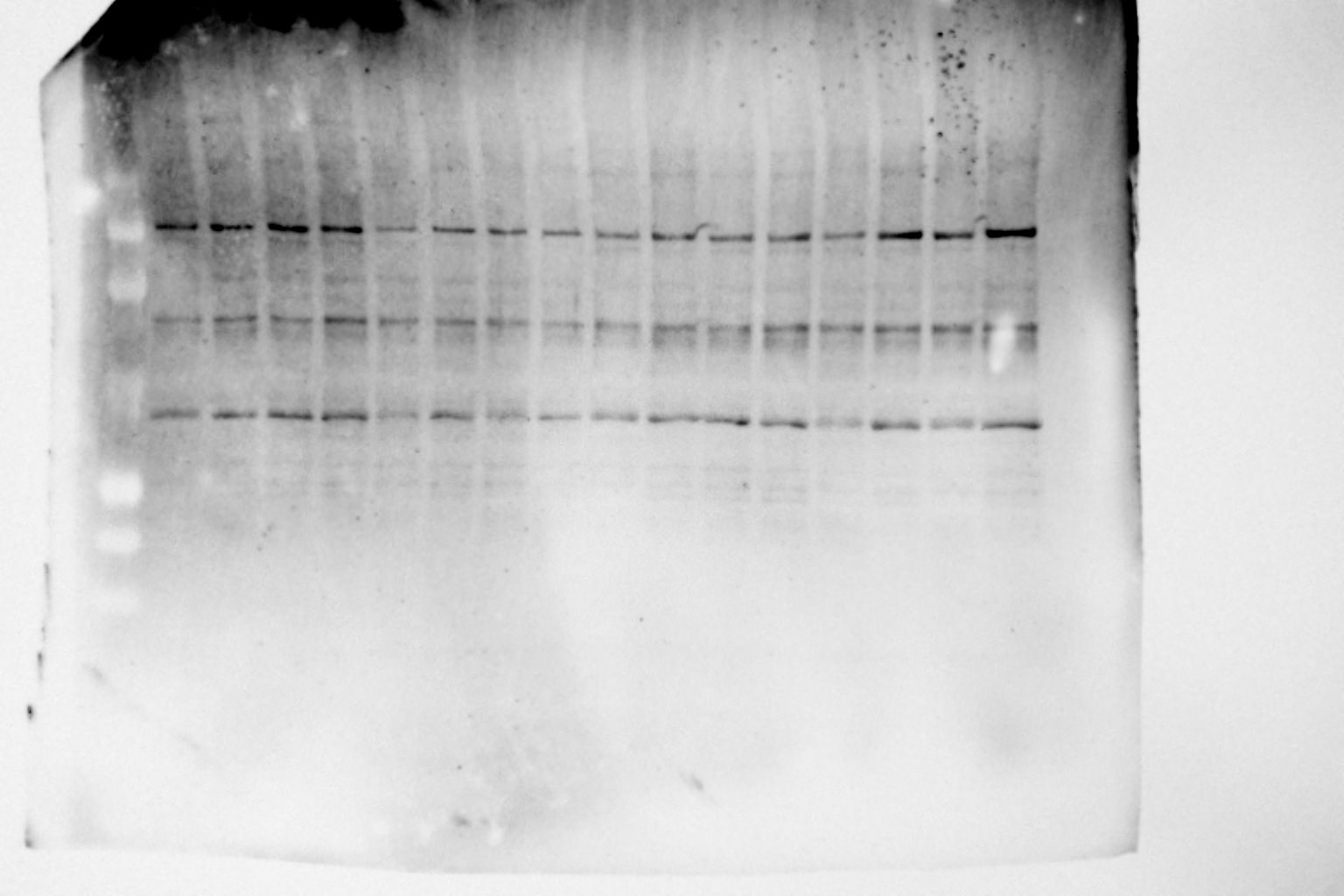

Supplement: Supplementary file 1 — Supplementary Material 1. [file 40959_2024_242_MOESM1_ESM.zip › Nrf2_Cyto.TIFF]

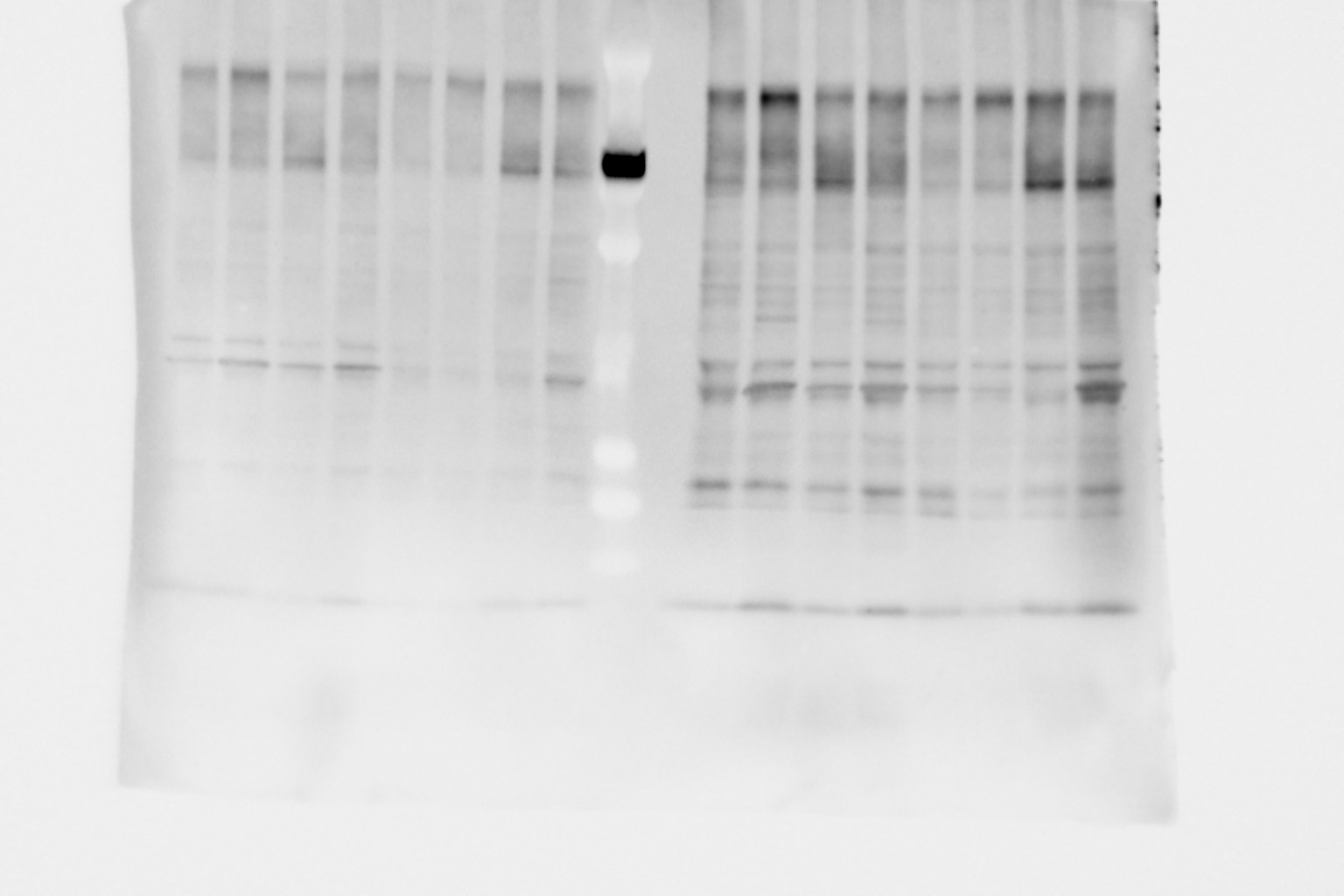

Supplement: Supplementary file 1 — Supplementary Material 1. [file 40959_2024_242_MOESM1_ESM.zip › Nrf2_histon_Nu.TIFF]

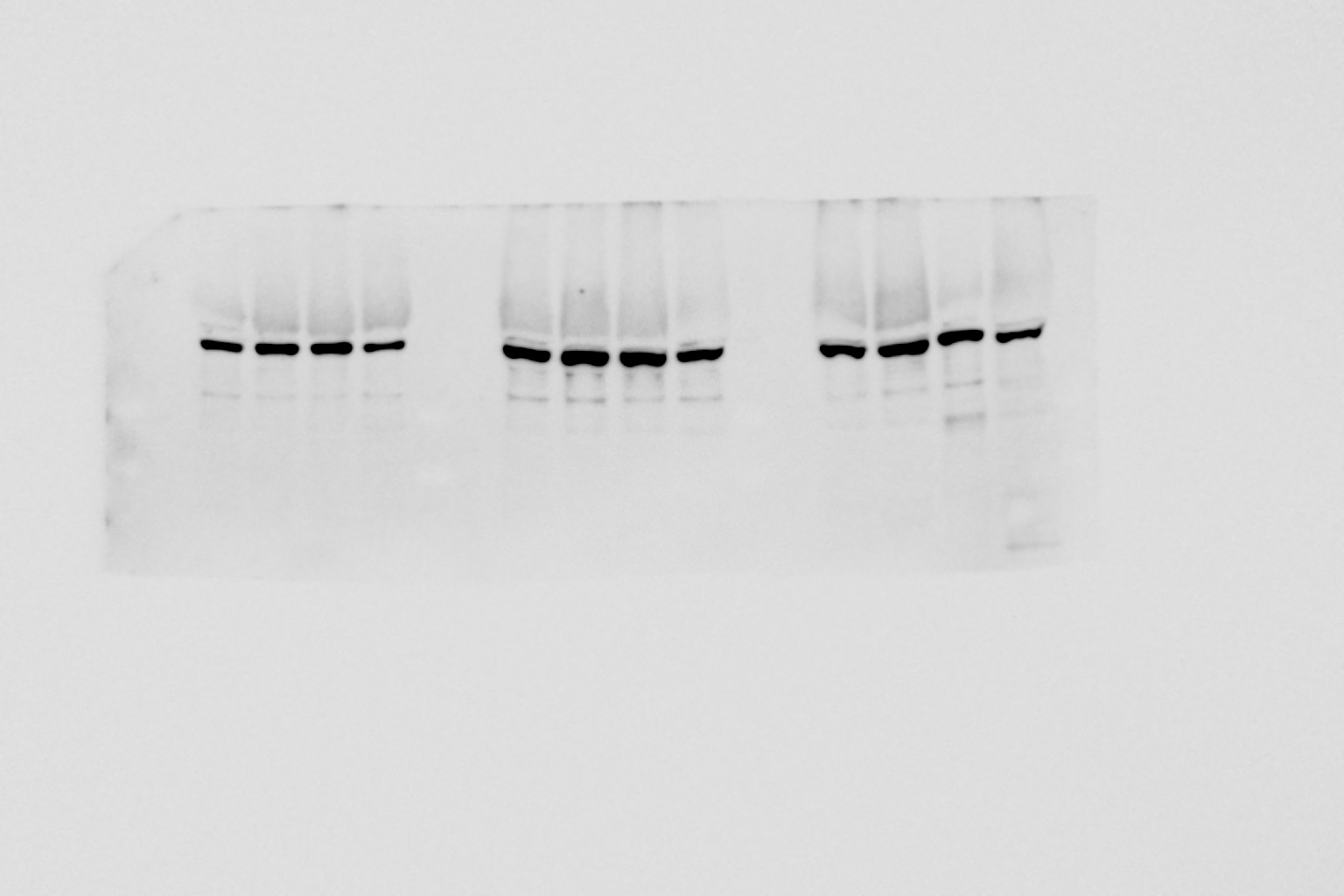

Supplement: Supplementary file 1 — Supplementary Material 1. [file 40959_2024_242_MOESM1_ESM.zip › TfR.revision.TIFF]
